# Physically crosslinked polyvinyl alcohol hydrogels as synthetic cartilage materials

**DOI:** 10.1080/07853890.2021.1896904

**Published:** 2021-09-28

**Authors:** S. Schweizer, I. Monteiro, A.S Oliveira, P. Nolasco, R. Colaço, A.P Serro

**Affiliations:** a Chemical Engineering Department, University of Reutlingen, Reutlingen, Germany; b CQE, Instituto Superior Técnico – University of Lisbon, Lisbon, Portugal; c IDMEC, Instituto Superior Técnico – University of Lisbon, Lisbon, Portugal; d Centro de Investigação Interdisciplinar Egas Moniz (CiiEM), Egas Moniz Cooperativa de Ensino Superior, Caparica, Portugal

## Abstract

**Introduction:**

Polyvinyl alcohol (PVA) hydrogels have been considered very promising materials for the replacement of cartilage tissues due to their biocompatibility, chemical resistance, swelling capacity, and tribological behaviour [1,2]. However, their mechanical properties are still far from those of articular cartilage. In the present work, some PVA hydrogels are prepared with different compositions and under different conditions, to obtain materials with superior physical and mechanical properties.

**Materials and methods:**

A 13.5% w/w PVA solution, prepared by dissolving the polymer (Mw 145,000 Da) in pure water for 20 h at 95 °C, was poured into Petri dishes and cooled to room temperature (8 h). Cast-drying (CD) (60 °C, 80% RH, 7 days) method was used to produce CD_PVA_ gels. Some of these were subsequently annealed for 30 min at 100 °C, giving rise to CD_PVA+A100_ samples. The freeze-thawing (FT) procedure (6 cycles of 16 h of freezing at −20 °C and 8 h of thawing at room temperature) was chosen to prepare FT_PVA_ and FT_PVA+PAA_ samples. For the latter case, polyacrylic acid (PAA, MW 100,000 Da) was added to the PVA solution in the ratio of 3:10 (w/w) in relation to the PVA. The materials were characterised in terms of water content, wettability (captive bubble method), microstructure (SEM) and mechanical performance (compression tests).

**Results:**

The CD gels presented lower water content and contact angles, a non-porous microstructure (see Figure 1), and higher rigidity than the FT samples. The annealing procedure slightly affected the studied properties of CD_PVA_, while the addition of PAA improved the water absorption of FT gels. **Discussion and conclusions:** The characteristics of PVA-based hydrogels can be easily tailored by adjusting the production method or combining PVA with other compounds in order to produce materials that best resemble human cartilage, and that can be used as substitutes for joint cartilage tissue.

**Figure 1.**
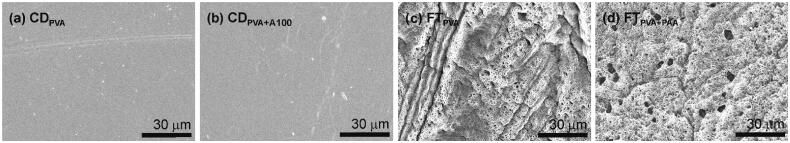
Microstructure of (a) CD_PVA_, (b) CD_PVA+A100_, (c) FT_PVA_**_,_** and (d) FT_PVA+PAA_.
